# In Vitro Inhibition of NFAT5-Mediated Induction of CCL2 in Hyperosmotic Conditions by Cyclosporine and Dexamethasone on Human HeLa-Modified Conjunctiva-Derived Cells

**DOI:** 10.1371/journal.pone.0159983

**Published:** 2016-08-03

**Authors:** Elise Warcoin, Christophe Baudouin, Carole Gard, Françoise Brignole-Baudouin

**Affiliations:** 1 Sorbonne Universités, UPMC Univ Paris 06, INSERM, CNRS, Institut de la Vision, Paris, France; 2 CHNO des Quinze-Vingts, Service Pharmacie, Paris, France; 3 CHNO des Quinze-Vingts, Service III, Paris, France; 4 Horus Pharma, Saint-Laurent du Var, France; 5 Faculté de Pharmacie de Paris, Univ Paris Descartes, Sorbonne Paris Cité, Paris, France; Save Sight Institute, AUSTRALIA

## Abstract

**Purpose:**

To investigate the pro-inflammatory intracellular mechanisms induced by an in vitro model of dry eye disease (DED) on a Hela-modified conjunctiva-derived cells in hyperosmolarity (HO) stress conditions. This study focused on CCL2 induction and explored the implications of the nuclear factor of activated T-cells 5 (NFAT5) as well as mitogen-activated protein kinases (MAPK) and nuclear factor kappa B (NFĸB). This work was completed by an analysis of the effects of cyclosporine A (CsA), dexamethasone (Dex) and doxycycline (Dox) on HO-induced CCL2 and NFAT5 induction.

**Methods:**

A human HeLa-modified conjunctiva-derived cell line was cultured in NaCl-hyperosmolar medium for various exposure times. Cellular viability, CCL2 secretion, NFAT5 and CCL2 gene expression, and intracytoplasmic NFAT5 were assessed using the Cell Titer Blue^®^ assay, enzyme-linked immunosorbent assay (ELISA), RT-qPCR and immunostaining, respectively. In selected experiments, inhibitors of MAPKs or NFκB, therapeutic agents or NFAT5 siRNAs were added before the hyperosmolar stimulations.

**Results:**

HO induced CCL2 secretion and expression as well as NFAT5 gene expression and translocation. Adding NFAT5-siRNA before hyperosmolar stimulation led to a complete inhibition of CCL2 induction and to a decrease in cellular viability. p38 MAPK (p38), c-Jun NH_2_-terminal kinase (JNK) and NFĸB inhibitors, CsA and Dex induced a partial inhibition of HO-induced CCL2, while Dox and extracellular signal-regulated kinase (ERK) inhibitor did not. Dex also induced a partial inhibition of HO-induced NFAT5 gene expression but not CsA or Dox.

**Conclusions:**

These in vitro results suggest a potential role of CCL2 in DED and highlight the crucial role of NFAT5 in the pro-inflammatory effect of HO on HeLa-modified conjunctiva-derived cells, a rarely studied cellular type. This inflammatory pathway involving NFAT5 and CCL2 could offer a promising target for developing new therapies to treat DED, warranting further investigations to fully grasp the complete intracellular mechanisms.

## Introduction

DED) is one of the most common ocular pathologies in the world, with a prevalence of 3–15% [1] in patients over the age of 50, although it is often underestimated because of its apparent harmlessness. However, patients with severe dry eye syndrome suffer from constant eye irritation symptoms as well as blurred and fluctuating vision [2,3] which can complicate daily tasks [4] and may in turn lead to anxiety and even depression [5]. DED is due to a dysfunction of the lachrymal functional unit resulting in decreased tear secretion and/or excessive evaporation of the aqueous tear phase. These effects then lead to an increase in tear film osmolarity, tear film instability and ultimately damages the ocular surface [6]. Tear HO and ocular surface inflammation are currently considered as the two key mechanisms underlying DED that maintain the vicious circle of the pathology on the ocular surface [1,7–9]. Clinical studies on dry eye patients reported an increase in pro-inflammatory cytokines and chemokines in tears and conjunctival cells such as interleukin (IL) -6, IL-8, TNF-α and IL-1β; a loss in conjunctival goblet cells [10,11]; and an increase in immune activation and infiltration in the conjunctiva [12–17]. To help understand the pathogenesis of DED, hyperosmolar conditions are often used because they reproduce the environment in contact with the ocular surface in the pathology. These experiments have shown that HO was responsible for ocular surface cell death [18,19], reactive oxygen species formation [20,21], activation of MAPKs such as p38, JNK and ERK [22–24] and increases in production of matrix metalloproteinases (MMP) [22], and pro-inflammatory cytokines such as IL-1β, TNF-α, IL-8, IL-6 and CCL2 [25–30].

The molecular mechanism that regulates the transcription and secretion of these pro-inflammatory actors under hyperosmolar conditions is poorly understood. Among the actors involved, CCL2, a potent chemoattractant protein that attracts monocytes to the inflammation site [31], and its receptor CCR2 have been identified as potentially important actors in DED. Indeed, Goyal et al. discovered that a topical antagonist of CCR2 improved dry eye symptoms in in vivo experiments [32]. On other cell types such as renal tubular epithelial cells and peritoneal mesothelial cells, the induction of pro-inflammatory cytokines such as CCL2 by osmotic stress has been observed to depend on the NFAT5 transcription factor, also called the tonicity response element-binding protein (TonEBP) [33,34]. HO is already known to induce NFAT5 translocation to promote cellular adaptation and survival from hypertonic stress [35]. Its activation in response to HO most particularly regulates the transcription of target genes that lead to the accumulation of compatible osmolytes inside cells, promoting better protection. Concerning ocular studies, a study on human limbal cells showed that IL-1β and TNF-α are induced in NaCl-induced hyperosmolar conditions via NFAT5 activation [36]. These accumulated observations suggest that NFAT5 could play a preponderant role in the inflammation induced by hypertonic challenge. Except for the study on cell death [18], no work has been specifically dedicated to the epithelial conjunctival cell inflammatory responses. However, conjunctival cells collected from dry eye patients express MHC class II antigen, HLA-DR, ICAM-1 and CCR5, confirming their involvement in the pathological process [15,37–39] and conjunctival inflammation was also reported in several in vivo models of the pathology [40,41].

Topical CsA is actually the only drug specifically formulated to treat dry eye syndrome and approved by regulatory medical agencies (FDA, EMA). Restasis^®^ (Allergan) and Ikervis^®^ (Santen) provide an alternative to artificial tears, Dex and Dox. CsA has immunosuppressive and anti-inflammatory effects, but its exact therapeutic mechanisms on the ocular surface of dry eye patients remains unclear.

The number of patients suffering from DED is set to grow as the world population ages. A better understanding of the pathophysiology of DED and the mechanism of CsA could help find new targets for treating this pathology and relieve millions of patients from permanent and painful discomfort.

**Fig 1:**
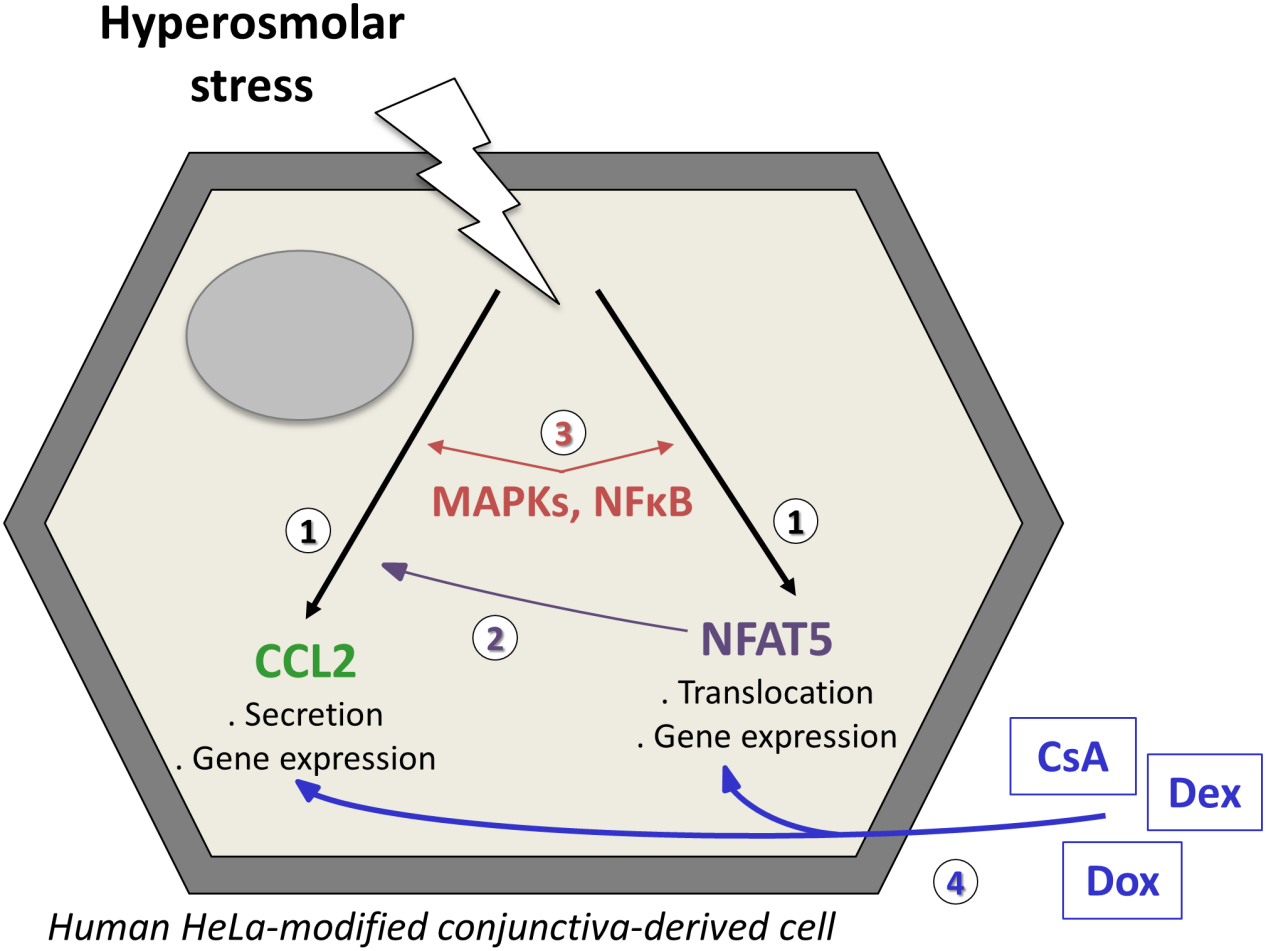
Schematic figure summarizing the hypotheses of the signaling mechanisms investigated in HeLa-modified conjunctiva-derived cells exposed to hyperosmolar conditions. 1) Is HO able to stimulate CCL2 or NFAT5? 2) Is NFAT5 involved in the CCL2 stimulation? 3) Are MAPKs or NFĸB implicated in the CCL2 and NFAT5 stimulations? 4) What are the effects of CsA, Dex or Dox on these stimulations?

Therefore, our aim was to analyze and characterize in vitro conjunctival CCL2 induction in a hyperosmolar model of dry eye and to determine the relationship between NFAT5 and CCL2 on HeLa-modified conjunctiva-derived cells. Moreover, we investigated intracellular signaling pathways such as MAPKs (p38, JNK and ERK) and the NFĸB transcription factor. We also investigated the effects of the three major anti-inflammatory therapeutic agents: CsA, Dex and Dox on CCL2 and NFAT5 induction by HO (Fig 1). These molecules are used in DED patients as an acute or chronic treatment, and understanding their anti-inflammatory mechanism on the desiccated ocular surface would help develop new targeted therapies.

## Materials and Methods

### Cell Line

The Wong Kilbourne derivative of the Chang (WKD) HeLa-modified conjunctiva-derived epithelial cell line (clone 1-5c-4, American Type Culture Collection [ATCC] certified cell line [CCL], 20.2) was cultured under classic conditions (moist atmosphere, 5% CO_2_, 37°C) in Dulbecco minimum essential medium supplemented with 10% fetal bovine serum, 1% glutamine (200 mM), 1% penicillin (10,000 units/mL) and streptomycin (10,000 μg/mL) for 24 h to reach either 80% confluence before hyperosmolar stimulations and to reach 50% confluence before transfection for siRNA experiments. All reagents for cellular culture were purchased from Gibco (Gibco, Life technologies, Carlsbad, CA, USA). This cell line has previously been used for toxicological in vitro studies and was shown to respond similarly to the IOBA conjunctival cell line [42]. Despite the criticism on this cell line, immortalized by contact with cervical cancer HeLa cells, we decided to use it after having confirmed the conjunctival phenotype by a cytokeratin 13 flow cytometry analysis [43] and because it does not require supplementation of hydrocortisone. Moreover, a study comparing gene expression profiles of conjunctival cell lines, including WKD cells, with primary cultured conjunctival epithelial cells and human conjunctival tissue, explained that these cell lines may still be used to investigate some specific cellular mechanisms like those related to inflammation, as human tissues and primary conjunctival cells are often difficult to obtain [44].

### Hyperosmolar Condition Preparation and Control

Each hyperosmolar medium was prepared by adding sodium chloride (Sigma-Aldrich, Saint Louis, MO, USA) to supplemented medium. All osmolarity values were assessed using an osmometer (Roebling 13DR, Berlin, Germany) including the supplemented medium, which was found at 340 mOsM (mosm.L^-1^) as expected from information provided by the manufacturer. We decided not to change this osmolarity level in order to keep the cell line in its regular medium even if this value is above the expected values of normal tear film, i.e., 302.2±8.3 mOsM, and in order to avoid any hypo-osmotic regulatory effects. In dry eye subjects, tear osmolarity is generally found at 336.4±22.3 mOsM [45] but some studies have reported higher tear hyperosmolarities that can reach 440 mosM [46,47] and spikes of 800–900 mOsM are thought to occur over the central cornea during tear film instability [8].

### Cell Treatments

For experiments that analyzed a range of hyperosmolar conditions, cells were treated with supplemented medium (340 mOsM) or with hyperosmolar medium ranging from 400 mOsM to 600 mOsM for 4 h before collecting cell lysates for real-time quantitative PCR (RT-qPCR), or 24 h before collecting supernatants for ELISA, performing the cell viability test or fixing cells for immunostaining. For the time-course experiments, cells were treated with medium (340 mOsM) or with hyperosmolar medium (500 mOsM) for durations ranging from 30 min to 24 h before collecting cell lysates for RT-qPCR, supernatants for ELISA or performing the cell viability test. This kind of protocol using a high osmolarity such as 500 mOsM during long duration as 24h on ocular surface cells is a common protocol often reported to study DED [19,36,48].

For the siRNA experiments, cells were treated with a mix of lipofectamine (Life Technologies, Carlsbad, CA, USA) and negative control siRNA (Life Technologies) or NFAT5 siRNA (Life Technologies) for 24 h, as recommended by the manufacturer. After this period of transfection, the cell medium was replaced by supplemented medium (340 mOsM) or hyperosmolar medium (500 mOsM) for 4 h before collecting cell lysates for RT-qPCR or 24 h before collecting supernatants for ELISA or performing the cell viability test.

For experiments testing the effects of therapeutic agents or cellular pathway inhibitors, cells were treated for 1 h with various dilutions of therapeutic agents. CsA was tested at 0.1, 1 and 10 μg/mL; the highest concentration of 10 μg/mL corresponding to 1/100 and 1/50 of Ikervis^®^ and Restasis^®^, respectively. Dex was tested at 10^−10^, 10^−8^ and 10^-6^M and Dox at 10 μg/mL. A mix of CsA 10 μg/mL and Dex 10–8 M was also tested. The inhibitors of p38 (SB203580), JNK (SP600125) and MEK/ERK (UO126) (inhibitor of MEK1 and hence of its downstream target ERK) were tested at 10 μM while the NFĸB inhibitor (PDTC) was tested at 50 μM. The concentrations tested were chosen regarding previous published studies [22,49,50] and after confirming their innocuity on HeLa-modified conjunctiva-derived cells with a cellular viability test, the CellTiter-Blue^®^assay (Promega, Madison, WI, USA). Therapeutic agents and inhibitors were all purchased from Sigma-Aldrich and were reconstituted with dimethylsulfoxide (DMSO) (Sigma-Aldrich) except for Dex that was reconstituted in water. They all were first diluted in DMSO and then in supplemented medium to achieve a final concentration of DMSO equal to 0.1%. The control condition for these experiments was DMSO 0.1% in supplemented medium. After a 1-h incubation, supplemented medium or hyperosmolar medium at 980 mOsM was added to induce a dilution of ¾ of the existing medium, leading to a final osmolarity of 340 mOsM and 500 mOsM, respectively. These conditions were applied for 4 h before collecting cell lysates for RT-qPCR, or 24 h before collecting supernatants for ELISA or performing the cell viability test. Pre-treating cells with therapeutic molecules or inhibitors before adding hyperosmolar condition is a classic protocol already reported on ocular surface cells [22,23,51].

For all these culture assays, we used one well for each condition concerning immunostaining (6-wells plate) or RTqPCR assays (24-wells plate) and at least two wells for ELISA and viability test (96-wells plate).

### Cell Viability Test

Cellular viability was analyzed using resazurin dye to measure the metabolic capacity of cells, which is an indicator of cell viability, using the CellTiter-Blue^®^assay (Promega). After an incubation period under the above-described stress conditions, CellTiter-Blue^®^ reagent was added to the cell culture following the protocol described by the manufacturer. Fluorescence intensity was then quantified using a microplate reader with an excitation wavelength of 560 nm and an emission wavelength of 590 nm (Infinite M1000, Tecan, Lyon, France). Fluorescence values were then normalized with respect to control cells considered as 100% viable.

### Enzyme-Linked Immunosorbent Assay

Enzyme-linked immunosorbent assays (ELISAs) for human CCL-2 were performed using the commercial DuoSet ELISA Development kit (R&D Systems, Minneapolis, MN, USA). After the incubation period under different stress conditions, cellular supernatants were collected, centrifuged to remove potential cellular fragments, and were stored at −80°C. ELISAs were performed on supernatants according to the manufacturer’s protocols. Absorbance was read at 450 nm using a microplate reader (Infinite M1000, Tecan) with a reference wavelength of 570 nm

### Immunostaining

Cells were grown on round sterile cover glasses (diameter, 14 mm; Menzel GmbH, Braunschweig, Germany). After the incubation period under different stress conditions, the cells were washed and fixed in 4% paraformaldehyde-PBS (Sigma-Aldrich). They were then permeabilized in a 0.3% Triton (Triton X-100, Sigma-Aldrich) solution for intracellular staining, followed by a 1% bovine serum albumin (Calbiochem, Merck Millipore, Darmstadt, Germany) incubation for 30 min and an overnight period with the primary antibody anti-NFAT5 at a final concentration of 2 μg.mL^-1^ (goat polyclonal antibody SC5499; Santa Cruz Biotechnology, Santa Cruz, CA, USA). Cells were then incubated for 1 h with the secondary antibody Alexa Fluor 488 rabbit anti-goat (Invitrogen, Life Technologies). Cover glasses were then mounted with Mountant PermaFluor^®^ (Thermo Fisher Scientific, Courtaboeuf, France) before observation with an epifluorescence microscope (Leica DM6000B, Rueil-Malmaison, France).

### RNA Extraction, Reverse Transcription, and Quantitative Real-Time PCR

After the incubation period under different stress conditions, the cells were washed and lysed, and their total RNA was extracted using a NucleoSpin RNA II extraction kit (Macherey-Nagel, Düren, Germany). RNA content was measured using a NanoDrop detector (ND-1000 spectrophotometer) and cDNA was synthesized from equal amounts of RNA using Multiscribe reverse transcriptase (TaqMan Reverse Transcription Reagents, Applied Biosystems, Life Technologies). Concentrations of each sample were adjusted to 5 ng/μL of cDNA. The reaction mixture containing 25 ng of cDNA per well was preheated at 95°C for 10 min, followed by 40 cycles (95°C/15 s and 60°C/1 min). Each assay was normalized by amplifying the housekeeping cDNA GAPDH (ID Hs9999905). Target cDNA was amplified using the 7300 Real-Time PCR system (Applied Biosystems, Life Technologies) with assays-on-demand primers for human CCL2 (Hs00234140) and NFAT5 (Hs00232437) (Applied Biosystems, Life Technologies). Changes in mRNA expression were calculated according to the 2-ΔΔCT method (CT, cycle threshold), with ΔCT = CT_target gene_-CT_gapdh_ and ΔΔCT = ΔCT_stimulated_-ΔCT_control_.

### Statistical Analyses

All experiments were performed at least three times: immunostainings and RTqPCR assays were performed on one well per condition, in three separated experiments. Viability tests and ELISA were performed at least in two wells per condition, in three separated experiments. Their conditions were compared using one-way analysis of variance (ANOVA) followed by Dunnett’s multiple comparison test, or two-way ANOVA followed by Sidak’s or Tukey’s multiple comparison test (GraphPad, GraphPad Software, La Jolla, CA, USA). Only *p*-values <0.05 were considered statistically significant.

## Results

### Hyperosmolar conditions, from 450 or 500 mOsM, Induced Cell Death in an Osmo-Dependent Manner, CCL2 Secretion and Gene Expression, and NFAT5 Gene Expression and Translocation in HeLa-Modified Conjunctiva-Derived Cells (Fig 2)

**Fig 2:**
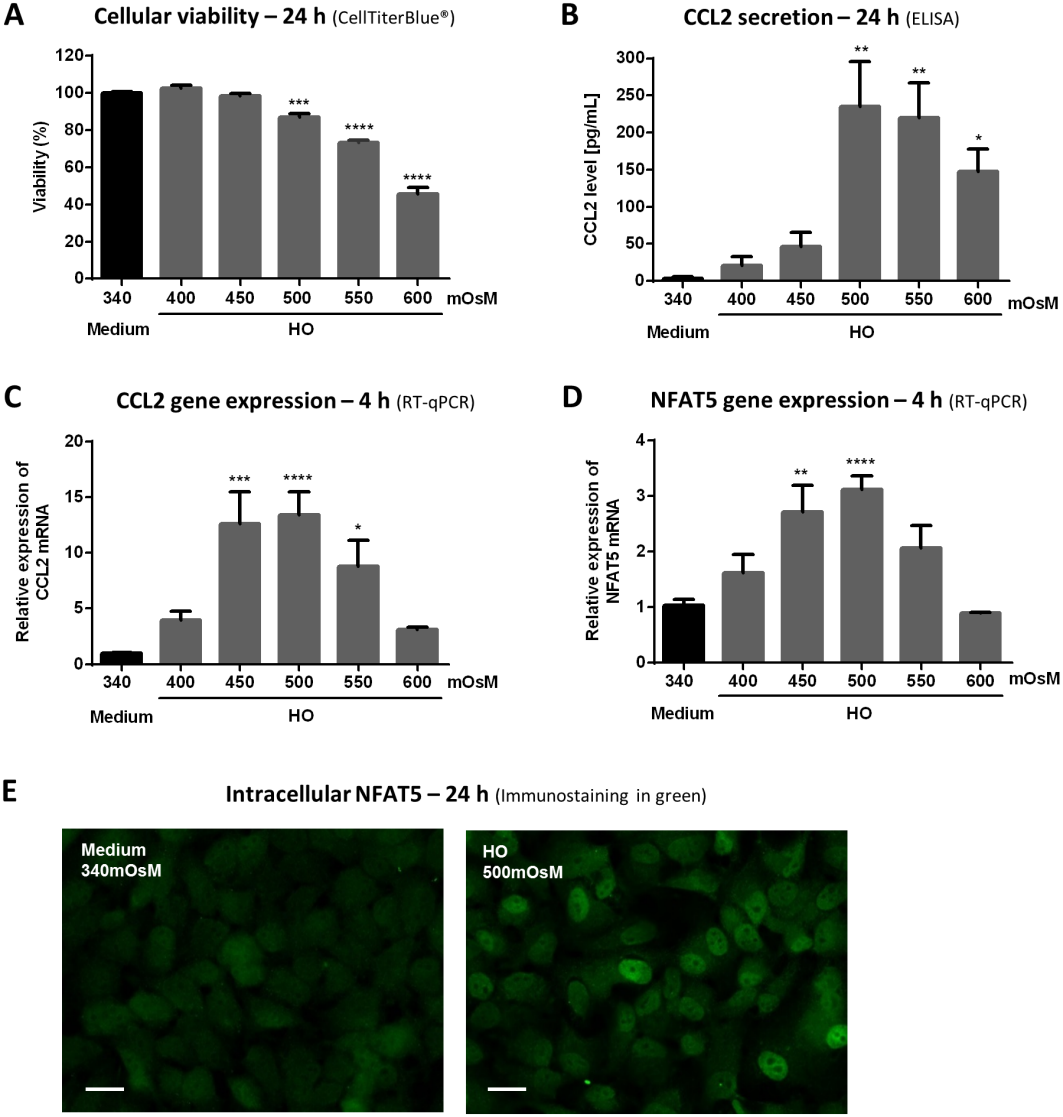
Hyperosmolar conditions, from 450 or 500 mOsM, induced cell death in an osmo-dependent manner, CCL2 secretion and gene expression, and NFAT5 gene expression and translocation in HeLa-modified conjunctiva-derived cells. Cells exposed to supplemented medium (340 mOsM) or different hyperosmolar conditions from 400 to 600 mOsM for 24 h were tested for cellular viability (A) with CellTiterBlue^®^ assay and CCL2 secretion (B) by ELISA, and cells treated for 4 h were lysed for mRNA expression by RT-qPCR to determine the CCL2 and NFAT5 gene expression (C, D). Data show mean ± SEM, *n* = 3 or more. Statistical analysis: one-way ANOVA followed by Dunnett's multiple comparison test. * *p* ≤ 0.05; ** *p* ≤ 0.01; *** *p* ≤ 0.001; **** *p* ≤ 0.0001, compared to 340 mOsM. Cells exposed to supplemented medium (340 mOsM) or hyperosmolar medium (500 mOsM) for 24 h were analyzed for NFAT5 translocation with NFAT5 immunofluorescent staining (E) in green. White scale bar: 20μm.

#### Cell death

After a 24-h treatment, conjunctival cellular viability significantly decreased in the 500-mOsM hyperosmolar condition, with 16% of cell viability loss compared to control (*p* ≤ 0.001) (Fig 2A). Higher hyperosmolar conditions, 550 and 600 mOsM, decreased viability even further, to values of 71 and 52%, respectively (*p* ≤ 0.0001).

#### CCL2 secretion and gene expression

Hyperosmolar conditions (500, 550 or 600 mOsM) applied for 24 h induced a significant secretion of CCL2 in supernatants by HeLa-modified conjunctiva-derived cells (235, 220, 148 pg/mL, respectively) (*p* ≤ 0.01 or *p* ≤ 0.05 compared to control cells at 3 pg/mL of CCL2 in supernatants), but no effect was detected for 400- and 450-mOsM conditions relative to the control osmolarity (Fig 2B).

CCL2 mRNA expression significantly increased in HO 450 mOsM, reaching a relative expression of 12.6 compared to the 340-mOsM control after 4 h (*p* ≤ 0.001) (Fig 2C). This relative fold expression was also significantly higher than the control, in 500- and 550-mOsM conditions (13.4 and 8.8, respectively). Neither the highest (600 mOsM) nor the lowest (400 mOsM) HO tested modified CCL2 mRNA expression compared to the control medium.

#### NFAT5 gene expression and nuclear translocation

Four hours in hypertonic conditions at 450 and 500 mOsM induced an increase in NFAT5 gene expression, 2.7- and 3.1-fold respectively, compared to the 340-mOsM condition (*p* ≤ 0.01 and *p* ≤ 0.0001, respectively) (Fig 2D). The immunostaining of NFAT5 showed the presence of NFAT5 both in the cytoplasm and the nuclei for control cells in 340 mOsM with a fine diffuse fluorescent staining (Fig 2E). Cells in hyperosmotic conditions of 500 mOsM for 24 h displayed an intense fluorescent staining in the nuclei, indicating the nuclear translocation of this transcription factor.

### Hyperosmolar Condition of 500 mOsM Induced Cell Death, Secretion and mRNA Expression of CCL2, and NFAT5 Gene Expression in HeLa-Modified Conjunctiva-Derived Cells in a Time-Dependent Manner (Fig 3)

**Fig 3:**
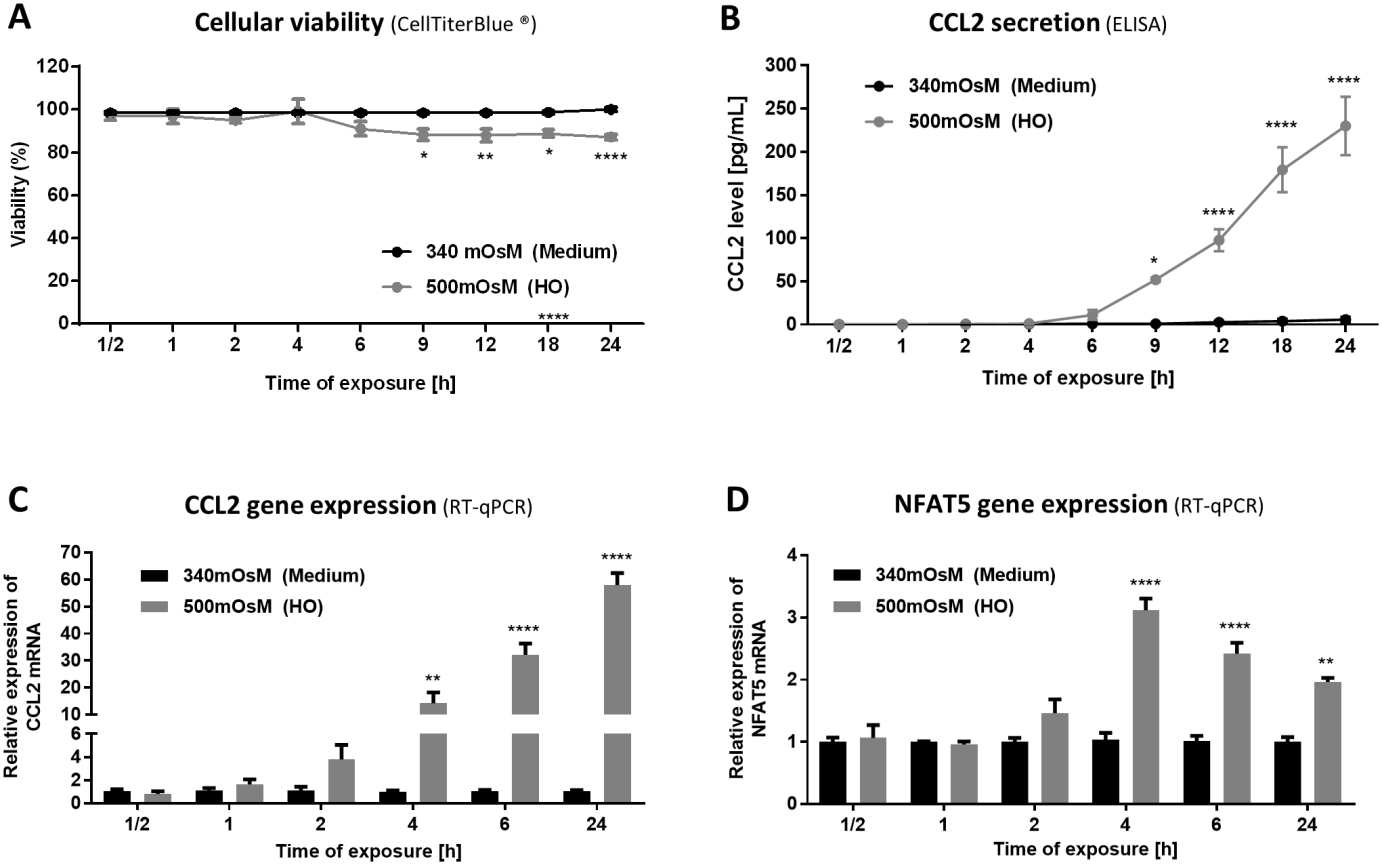
Hyperosmolar condition of 500 mOsM induced cell death, secretion and gene expression of CCL2 and NFAT5 gene expression in HeLa-modified conjunctiva-derived cells in a time-dependent manner. Cells exposed to supplemented medium (340 mOsM) or the hyperosmolar condition (500 mOsM) for different times from 30 min to 24 h were tested for cellular viability (A) with CellTiterBlue^®^ assay, CCL2 secretion (B) with ELISA and were lysed for mRNA expression using RT-qPCR to determine CCL2 and NFAT5 gene expression (C, D). Data showing mean ± SEM, *n* = 3 or more. Statistical analysis: two-way ANOVA followed by Sidak’s multiple comparison test. * *p* ≤ 0.05; ** *p* ≤ 0.01; *** *p* ≤ 0.001; **** *p* ≤ 0.0001, compared to 340 mOsM at the same time.

#### Cell death

HeLa-modified conjunctiva-derived cells in a 500-mOsM medium started to significantly decrease their viability only after 9 h of stress (88% viability) (Fig 3A). The viability continued to decrease until 24 h of stress.

#### CCL2 secretion an gene expression

Similarly, CCL2 secretion significantly increased after 9 h in 500 mOsM (1 pg/mL vs 52 pg/mL) (*p* ≤ 0.05) (Fig 3B). CCL2 secretion increased further to concentrations of 98 pg/mL, 180 pg/mL and 235 mg/mL after 12, 18 and 24 h of hyperosmolar treatment (*p* ≤ 0.0001 compared to control cells).

CCL2 gene expression was also enhanced by the 500-mOsM medium with CCL2 mRNA expression increasing 14-fold compared to the 340-mOsM control after only 4 h in hyperosmolar conditions (Fig 3C). The mRNA expression increased 32-fold after 6 h and 58-fold after 24 h (*p* ≤ 0.0001 for both conditions).

#### NFAT5 gene expression

NFAT5 gene expression increased 3.1-fold compared to control cells after 4 h in hyperosmolar conditions (*p* ≤ 0.0001). NFAT5 mRNA expression significantly increased 2.4-fold and 2.0-fold following 6 and 24 h of treatment, respectively (Fig 3D).

### NFAT5 siRNA Increased HO-Induced Cell Death and Inhibited HO-Induced CCL2 Secretion and Gene Expression in HeLa-Modified Conjunctiva-Derived Cells (Fig 4)

**Fig 4:**
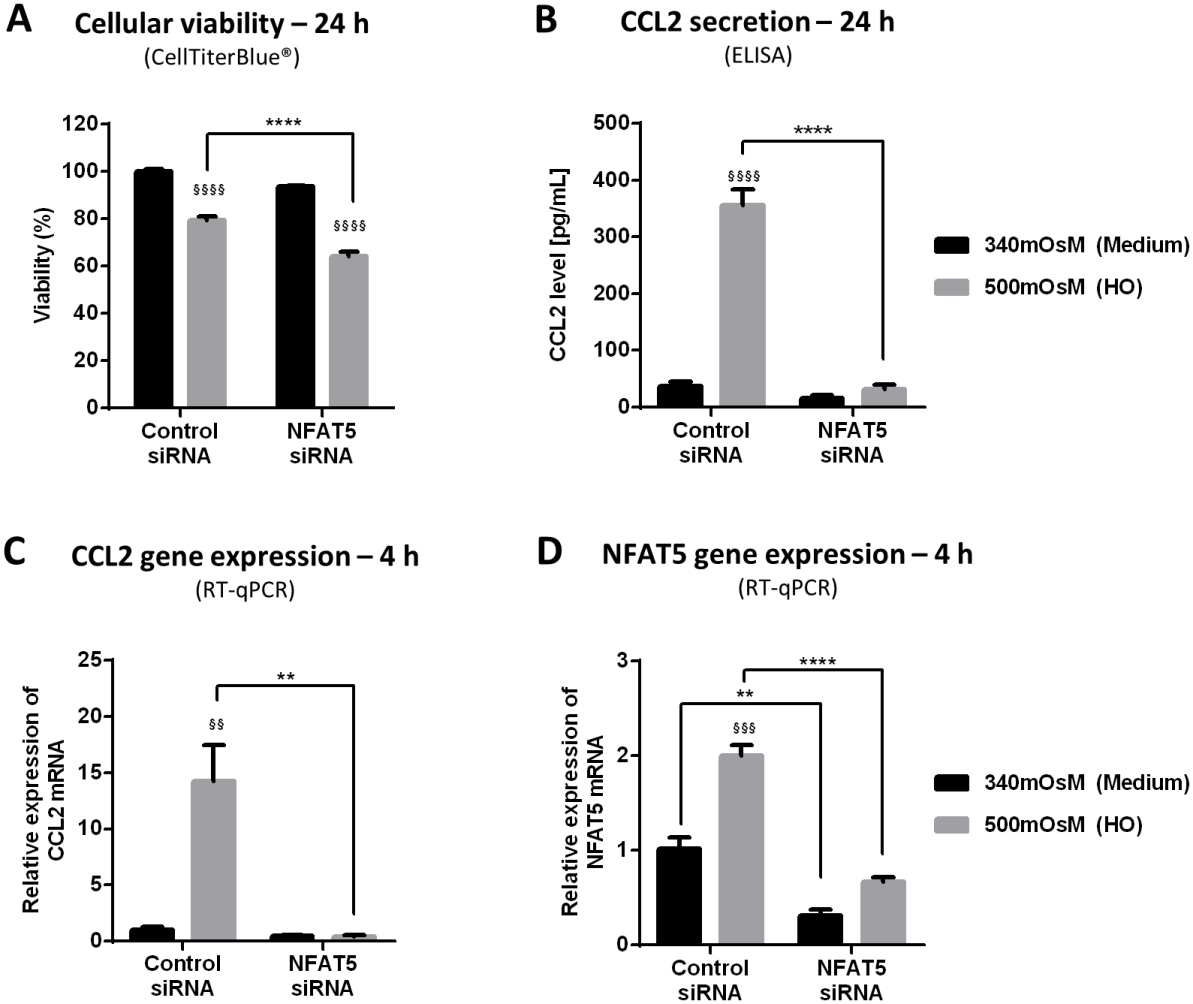
NFAT5 siRNA increased HO-induced cell death and inhibited HO-induced CCL2 secretion and CCL2 and NFAT5 gene expression in HeLa-modified conjunctiva-derived cells. Cells treated for 24 h with negative control siRNA or NFAT5 siRNA were then exposed to supplemented medium (340 mOsM) or hyperosmolar condition (500 mOsM). Cells exposed for 24 h were tested for cellular viability (A) with CellTiterBlue^®^ assay, CCL2 secretion (B) by ELISA and cells exposed for 4 h were lysed for mRNA expression using RT-qPCR to determine the CCL2 and NFAT5 gene expression (C, D). Data showing mean ± SEM, *n* = 3 or more. Statistical analysis: two-way ANOVA followed by Tukey’s multiple comparison test. § *p* ≤ 0.05; §§ *p* ≤ 0.01; §§§ *p* ≤ 0.001; §§§ *p* ≤ 0.0001, compared to respective 340 mOsM. * *p* ≤ 0.05; ** *p* ≤ 0.01; *** *p* ≤ 0.001; **** *p* ≤ 0.0001, compared to respective control siRNA.

#### Cell death

The cell viability test did not reveal any toxic effect when using NFAT5 siRNA versus control siRNA in basal conditions of 340 mOsM (Fig 4A). However, in 500-mOsM hyperosmolar medium, the viability of cells treated with NFAT5 siRNA was significantly lower than cells treated with control siRNA (64% versus 79%).

#### CCL2 secretion and gene expression

Concerning the CCL2 secretion in 340 mOsM, control and NFAT5 siRNAs did not induce significantly different effects, with a low level of CCL2 in supernatant (37 and 16 pg/mL, respectively) (Fig 4B). In hyperosmolar conditions, adding NFAT5 siRNA induced a complete inhibition of HO-induced CCL2 secretion: HO induced a CCL2 concentration of 356 pg/mL in supernatant of cells treated with control siRNA and CCL2 concentration reached 32 pg/mL with NFAT5 siRNA (*p* ≤ 0.0001). This low concentration did not significantly differ from that observed with NFAT5 siRNA in 340-mOsM conditions equal to 37 pg/mL (statistical analysis not shown).

The same inhibition profile was observed with CCL2 gene expression. The CCL2 mRNA expression with control siRNA increased 14.3-fold in 500-mOsM conditions compared to 340-msOsM conditions (Fig 4C). Treating cells with NFAT5 siRNA reduced this CCL2 mRNA expression to a value similar to those observed in basal conditions (0.4 vs 0.5).

#### NFAT5 gene expression

The specificity of the NFAT5 siRNA used was confirmed by RT-qPCR of NFAT5 (Fig 4D). Treating cells with NFAT5 siRNA significantly reduced NFAT5 mRNA expression compared to cells treated with control siRNA (0.3 vs 1.0 for 340 mOsM and 0.7 vs 2.0 for 500 mOsM).

The lack of toxicity of siRNAs was also verified using the CellTiter-Blue viability test. It showed that control siRNA and NFAT5 siRNA had no effect on the viability of HeLa-modified conjunctiva-derived cells compared to cells without siRNA in classic medium (data not shown).

### Effects of a p38 Inhibitor (SB203580), a JNK Inhibitor (SP600125), a MEK/ERK Inhibitor (U0126) and a NFκB Inhibitor (PDTC) on HO-Induced CCL2 Secretion, and CCL2 and NFAT5 Gene Expression in HeLa-Modified Conjunctiva-Derived Cells (Fig 5)

**Fig 5:**
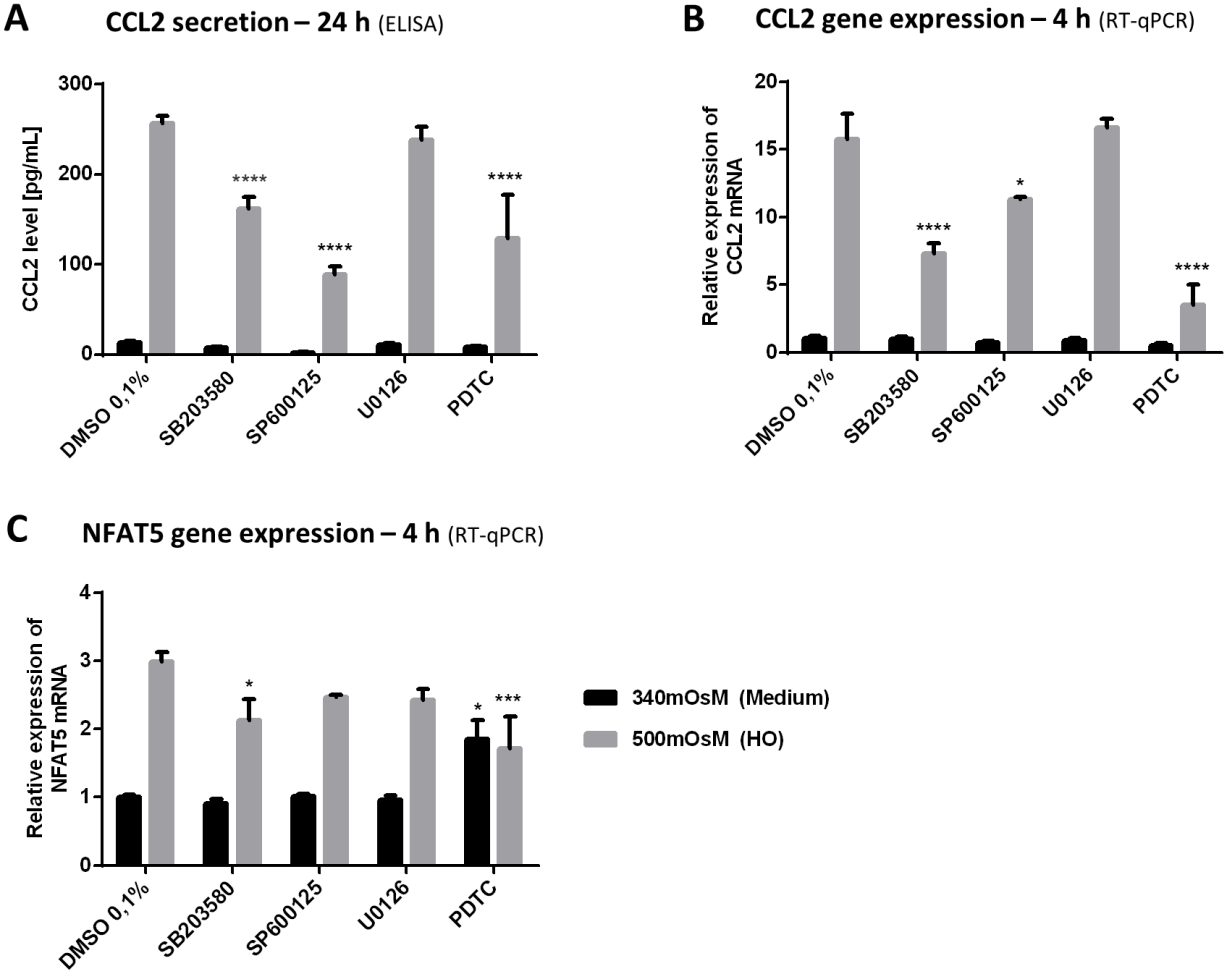
Effects of a p38 inhibitor (SB203580), a JNK inhibitor (SP600125), a MEK/ERK inhibitor (U0126) and a NFκB inhibitor (PDTC) on HO-induced CCL2 secretion and CCL2 and NFAT5 gene expression in HeLa-modified conjunctiva-derived cells. Cells were treated for 1 h with DMSO 0.1%, SB203580 10 μM, SP600125 10 μM, U0126 10 μM or PDTC 50 μM before adding supplemented medium 340 mOsM or hyperosmolar medium to reach 500 mOsM. Cells exposed to medium or HO for 24 h were tested for CCL2 secretion (A) by ELISA and cells exposed for 4 h were lysed for mRNA expression using RT-qPCR to determine the CCL2 and NFAT5 gene expression (B, C). Data showing mean ± SEM, *n* = 3 or more. Statistical analysis: two-way ANOVA followed by Tukey’s multiple comparison test. * *p* ≤ 0.05; ** *p* ≤ 0.01; *** *p* ≤ 0.001; **** *p* ≤ 0.0001, compared to respective DMSO 0.1%.

#### CCL2 secretion and gene expression

In hyperosmolar 500-mOsM conditions for 24 h, the control DMSO 0.1% induced CCL2 secretion in supernatants (257 pg/mL) (Fig 5A). This secretion significantly decreased for cells pretreated with the inhibitors SB203580, SP600125 and PDTC (162, 89, 130 pg/mL, respectively; *p* ≤ 0.0001 for the three conditions). Treating cells with U0126 had no effect on this secretion.

The same CCL2 mRNA inhibition profile was observed in hyperosmolar conditions following addition of the inhibitors SB203580, SP600125 and PDTC with a 7.4-, 11.3- and 3.5-fold mRNA increase, respectively, compared to the 18.4-fold increase for DMSO (Fig 5B). As observed during CCL2 secretion assays, U0126 had no effect on HO-induced CCL2 mRNA expression.

Under 340-mOsM basal conditions, the compounds tested had no effect on either the production of CCL2 by HeLa-modified conjunctiva-derived cells or its gene expression.

#### NFAT5 gene expression

Treating cells with PDTC increased NFAT5 gene expression 1.9-fold under basal 340-mOsM conditions (Fig 5C). A 3.0-fold increase in NFAT5 expression was recorded under hyperosmolar conditions containing DMSO. NFAT5 expression in 500-mOsM conditions increased more moderately when SB203580 and PDTC were added to medium (2.1- and 1.7-fold increase, respectively) contrary to SP600125 and U0126, which had no effect at all.

None of the compounds tested had a negative effect on cellular viability compared to their respective control conditions: DMSO 0.1% in 340 mOsM or in 500 mOsM (data not shown).

### Effects of CsA, Dex and Dox on HO-Induced CCL2 Secretion as well as CCL2 and NFAT5 Gene Expressions in HeLa-Modified Conjunctiva-Derived Cells (Fig 6)

**Fig 6:**
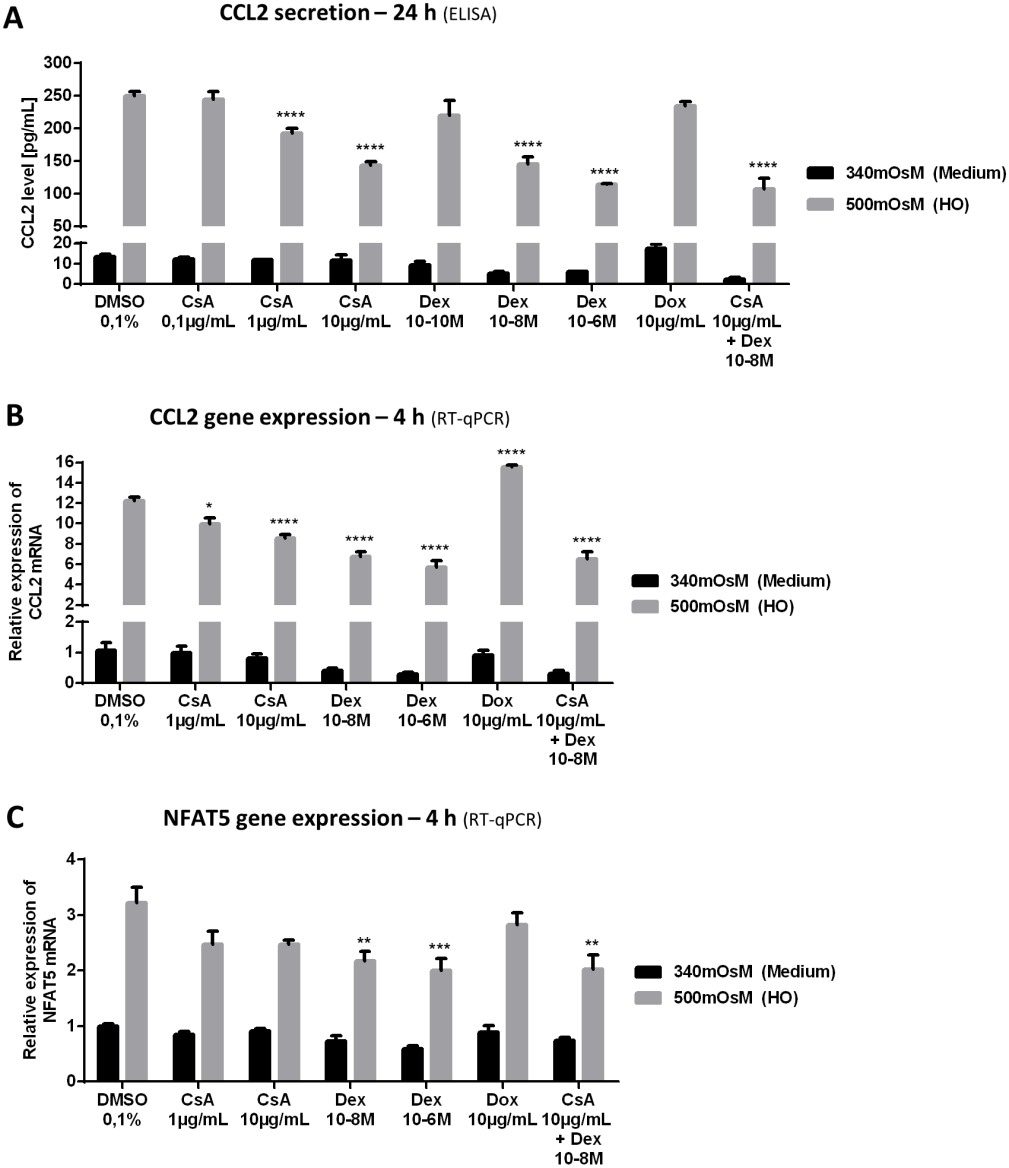
Effects of CsA, Dex and Dox on HO-induced CCL2 secretion and CCL2 and NFAT5 gene expression in HeLa-modified conjunctiva-derived cells. Cells were treated for 1 h with DMSO 0.1%, CsA (0.1, 1 and 10 μg/mL), Dex (10^−10^, 10^−8^ and 10^-6^M), Dox 10 μg/mL or a mix of CsA 10 μg/mL and Dex 10^-8^M before adding supplemented medium 340 mOsM or HO to reach 500 mOsM. Cells exposed to medium or HO for 24 h were tested for CCL2 secretion (A) by ELISA and cells exposed for 4 h were lysed for mRNA expression using RT-qPCR to determine the CCL2 and NFAT5 gene expression (B, C). Data showing mean ± SEM, *n* = 3 or more. Statistical analysis: two-way ANOVA followed by Tukey’s multiple comparison test. * *p* ≤ 0.05; ** *p* ≤ 0.01; *** *p* ≤ 0.001; **** *p* ≤ 0.0001, compared to respective DMSO 0.1%.

#### CCL2 secretion and gene expression

After 24 h in 500-mOsM conditions, CCL2 secretion with control DMSO 0.1% reached 250 pg/mL, whereas after 4 h, CCL2 mRNA expression increased 12.2-fold (Fig 6A and 6B). Both these effects were significantly inhibited in a concentration-dependent way by CsA and Dex. CsA 1 and 10 μg/mL decreased CCL2 levels to 193 and 144 pg/mL, respectively (*p* ≤ 0.0001 compared to DMSO), and lowered the increase in CCL2 expression to 10.0-fold and 8.6-fold, respectively. The lowest dose tested, 0.1 μg/mL, had no observable impact on CCL2 levels. The lowest dose of Dex tested (10^-10^M) also had no effect on CCL2 induction, but Dex 10^−8^ and 10^-6^M had a significant inhibitory effect on CCL2 secretion (145 and 114 pg/mL, respectively, *p* ≤ 0.0001 compared to DMSO) and on CCL2 mRNA expression (increasing it 6.7-fold and 5.7-fold, respectively, *p* ≤ 0.0001 compared to DMSO) in 500-mOsM conditions. In contrast, Dox had no inhibitory effect on CCL2 secretion induced by HO and even led to a significant increase in HO-induced CCL2 gene expression compared to DMSO (15.6-fold, *p* ≤ 0.0001).

#### NFAT5 gene expression

HO conditions increased NFAT5 mRNA expression 3.2-fold. This HO-induced increase was significantly inhibited by Dex 10^−8^ and 10^-6^M (2.2-fold and 2.0-fold, respectively) (Fig 6C). CsA and Dox induced no significant effect on NFAT5 gene expression.

Mixing Dex and CsA induced no effect that differed significantly from each of the conditions alone (statistics not shown; two-way ANOVA followed by Tukey’s multiple comparison test). Under 340-mOsM conditions, none of the compounds tested had any effect on the production and gene expression of CCL2, or on NFAT5 gene expression by HeLa-modified conjunctiva-derived cells. None of the compounds tested affected cellular viability compared to DMSO 0.1% conditions under 340 mOsM or 500 mOsM, respectively (data not shown).

## Discussion

Despite its worldwide high prevalence and its significant impact on everyday life for patients, DED actually has few symptomatic and curative treatments. It is therefore important to improve the understanding of the pathogenesis mechanisms with the ultimate goal of developing new targeted therapeutic strategies. Conjunctiva could be an important tissue to study as it covers the major part of the ocular surface and is the site where inflammatory reactions take place. Moreover, conjunctival cells themselves are able to secrete inflammatory cytokines and to participate in inflammatory processes [52]. Therefore, we used a HeLa-modified cell line derived from conjunctiva in a hyperosmolar NaCl-induced in vitro model of dry eye. We wished to characterize the mechanism of the induction of the CCL2 pro-inflammatory chemokine through cellular pathways, such as MAPK and NFĸB, with a focus on the NFAT5 transcription factor. We also investigated the effects of different drugs used in DED, among which CsA, on CCL2 and NFAT5 induction.

We demonstrated that HeLa-modified conjunctiva-derived cells respond to hyperosmotic stress by inducing gene expression and secretion of CCL2, just as corneal cells or other cell types do [53,54]. This induction is dependent on the osmolarity level, and with the highest range of osmolarity, this induction decreases, probably due to the substantial cell death that occurs under these conditions. The lowest osmolarity tested that induced both secretion and gene expression of CCL2 was 500 mOsM, a classical osmolarity level used in previous studies on ocular surface cells [55]. Our results tend to show that HO induces CCL2 by stimulating its gene expression and that there is no preformed stock of this chemokine in these cells, as under hyperosmolar condition, gene expression of CCL2 was induced before its secretion. By secreting this pro-inflammatory chemokine, conjunctival cells could therefore be responsible for the attraction of immune cells, namely monocytes/macrophages that will further nourish the inflammatory process on the ocular surface. Blocking this inflammatory pathway could then potentially be of therapeutic interest, as Goyal et al. have demonstrated in an in vivo model of dry eye [32]. Indeed, animals with dry eye receiving CCR2 antagonist presented a decrease in corneal alterations and in pro-inflammatory cytokines TNF-α and IL-1β on the ocular surface, confirming the importance of CCL2 in the pathology.

CsA displayed a concentration-dependent inhibitory effect on the HO-induced CCL2 production in our model. This was also observed with Dex, an effect previously reported in a study on corneal cells [29]. Conversely, Dox induced an elevation on CCL2 gene expression. Reported inhibitory effects of anti-inflammatory drugs on CCL2 induction are various. Regarding only studies on ocular cells, Dex was reported to inhibit CCL2 induced by IL-1β on retinal cells [56] but not on corneal cells [49]. In retinal epithelial cells, CsA has inhibited the CCL2 induction caused by TNFα but not by IL-1β [56]. In lung epithelial cells, Dox inhibited CCL2 production induced by a mix of cytokines [57]. In addition, we observed that in spite of having different mechanisms of action, CsA and Dex inhibit CCL2 induction probably through the same molecular pathway, since combining these two molecules did not result in a stronger inhibitory effect. It is noteworthy that the drug concentrations tested are lower than those actually present in eyedrops. Our results further highlight that the CCL2 induction pathway could represent an advantageous therapeutic target, as the anti-inflammatory effect of CsA and Dex observed in DED patients could partly be explained by its inhibition. It is therefore important to understand which molecular actors of this pathway lead to CCL2 secretion.

The three MAPKs, p38, JNK and ERK, as well as the transcription factor NFκB are activated by HO in several types of mammalian cells [33,58–61]. Previous studies on corneal cells showed that NaCl-induced HO activates p38 [51,62], JNK [19,22,51,63,64] and ERK [19] as well as NFĸB [65], and a recent study showed that HO activates JNK on primary conjunctival cells as well [66]. In our experimental conditions, we demonstrated that CCL2 stimulation by HO is related to p38, JNK and NFĸB but not to ERK. These results confirmed those of other studies conducted on corneal cells, which found that some effects of HO were directly linked to these pathways: induction of MMP [22], apoptosis [19] and induction of cytokine secretion such as IL-1β [50]. These pathways are therefore potential factors to consider for developing new drugs for DED and they are known to be repressed by CsA and Dex, including in ocular cell studies [29,67,68].

HO is known to activate NFAT5, a transcription factor that plays a key role in osmoprotection by driving osmoprotective gene expression, thus promoting cell survival and protecting cells from the deleterious effects of shrinkage. In addition to this fundamental role, it has a wider range of functions, such as inflammatory cytokine production [69,70] or growth factor stimulation [71]. In our experiments, we observed that HO induces NFAT5 translocation and mRNA expression, and we demonstrated that the CCL2 stimulation induced by HO is entirely mediated by NFAT5. Lee et al. showed that, in limbal cells, the induction of IL-1β and TNF-α by HO was also related to NFAT5 [36] and a relation between HO, CCL2 and NFAT5 has been reported in rat kidney cells and mesothelial cells [33,34]. In our experiments, we also confirmed the protective effect of NFAT5 on cell survival under hyperosmolar stress. These findings on HeLa-modified conjunctiva-derived cells highlight the potentially crucial role of NFAT5 in dry eye syndrome and the necessity to further investigate its mechanisms and effects in ocular cells. We also determined that the HO-induced NFAT5 expression was linked to p38 activity and that NFκB has an important dual role in NFAT5 expression, with a stimulatory effect in basal conditions and an inhibitory effect in HO. Another study reported that NFAT5 modulates NFκB activity in hyperosmolar conditions [72], confirming the important interaction between these two actors activated by hyperosmolar stress. We showed that Dex was able to decrease HO-induced NFAT5 mRNA expression. This effect could explain its inhibitory action on HO-induced CCL2 stimulation. CsA also induces a decrease on it but in a non-statistically significant manner. Other studies on corneal or collecting duct cells have shown a lack of effect of CsA on NFAT5 expression [73,74]. Overall, our results tend to show similar effects induced by CsA and Dex on HO-induced response in HeLa-modified conjunctiva-derived cells, especially on CCL2 induction.

All these results obtained with HeLa-modified-WKD cells should be compared and confirmed with primary human conjunctival cells. Nevertheless, our results highlight the potentially important role of conjunctival cells in the response of the ocular surface to a modification of the tear film. They are able to secrete pro-inflammatory mediators that could participate in the vicious circle of DED by interfering with the inflammatory process stimulated by tear film HO. The inflammatory cellular pathway of NFAT5 translocation, a typical feature of hyperosmolar stress, leading to CCL2 secretion through other factors such as MAPKs and NFκB, constitutes a biological cascade with potentially promising new targets of interest for DED therapy.

## Supporting Information

S1 Table Raw data from Fig 2. (XLSX)

S2 Table Raw data from Fig 3. (XLSX)

S3 Table Raw data from Fig 4. (XLSX)

S4 Table Raw data from Fig 5. (XLSX)

S5 Table Raw data from Fig 6. (XLSX)
